# Editorial: Disambiguating pubertal status from pubertal timing during adolescence: A renewed call for improved construct definition and measurement

**DOI:** 10.1111/jcpp.70172

**Published:** 2026-05-29

**Authors:** Kelly L. Klump, Alaina M. Di Dio, Kristen M. Culbert

**Affiliations:** ^1^ Department of Psychology Michigan State University East Lansing MI USA; ^2^ Department of Psychology and Neuroscience University of Colorado Boulder Boulder CO USA; ^3^ Institute for Behavioral Genetics University of Colorado Boulder Boulder CO USA

**Keywords:** Puberty, pubertal status, pubertal timing, measurement, adolescence, developmental psychopathology

## Abstract

There have been numerous past calls for studies to examine pubertal development, rather than just age, in developmental research. These calls are timely and appropriate given the significant effects of pubertal development on a range of internalizing (e.g., eating disorders, depression) and externalizing (e.g., antisocial behavior) disorders. However, with this increased attention, some ambiguity in puberty constructs have crept into this important area of research. Previously clear lines between pubertal status versus pubertal timing have become blurred. In this Editorial, we define pubertal status and timing, describe how definitions and measurement models can confound these constructs, and propose new language for describing pubertal status and timing effects that may help establish clearer lines between these pubertal processes. We hope this piece will be a catalyst for more research and a testing of our lexical and empirical suggestions that are aimed at moving the field of developmental psychopathology forward in this critical area of adolescent health.

There have been numerous past calls for studies to examine pubertal development, rather than just age, in developmental research (Beltz et al., 2026; Berenbaum, Beltz, & Corley, 2015; Dorn, Dahl, Woodward, & Biro, 2006; Klump, 2022). These calls are appropriate given the significant effects of pubertal development on internalizing (e.g., eating disorders, depression) and externalizing (e.g., antisocial behavior) (Goering & Mrug, 2022; Klump, 2013; Ullsperger & Nikolas, 2017) disorders. New research in this issue of the *Journal of Child Psychology and Psychiatry* (see Xu, Wang, Chen, Xiong, & Cheng, 2026) underscores the importance of puberty for positive youth development as well.

However, with increased attention, some ambiguity in puberty constructs has emerged. Previously clear lines between pubertal status and pubertal timing have become blurred. This blurring is the result of good intentions; researchers aim to fully explore status and timing using any samples available. However, it comes at a cost in terms of fully understanding how and if pubertal status and timing influence youth mental health. In this Editorial, we define pubertal status and timing, describe how these constructs are often confounded/confused, and propose strategies for establishing clearer lines between them.

We define pubertal status and timing, describe how these constructs are often confounded/confused, and propose strategies for establishing clearer lines between them.

## Definitions

### Pubertal status

Pubertal status is the level of physical maturation during the childhood‐to‐sexual maturity transition. Development of secondary sex characteristics (e.g., breast development, menses in girls; body hair, voice changes in boys) has most commonly been used to stage youth from pre‐puberty (no physical changes) to post‐puberty (physical maturation is complete) (Dorn et al., 2006). Status is most commonly assessed using self‐/parent‐reports (e.g., on the Pubertal Development Scale (PDS)) or clinician assessments (e.g., Tanner staging) (Dorn et al., 2006). Researchers are often concerned about disentangling the effects of status from age, as more advanced pubertal development typically occurs with more advanced age, and age itself is a marker of biopsychosocial changes that independently impact youth development (Berenbaum et al., 2015). Age is often addressed by covarying age in models of status or examining differences in status in youth who are the same age.

### Pubertal timing

Pubertal timing is pubertal status relative to same‐age peers (Ullsperger & Nikolas, 2017). Timing is categorized as early (i.e., maturing earlier than peers), on time (i.e., maturing at the same time), or late (i.e., maturing later than peers), and is defined using both objective and subjective indices. Objective timing assesses the timing of observable markers of maturation compared with a normative group of same‐age peers (e.g., population mean age of menses). By contrast, subjective timing assesses youth perceptions of pubertal maturation in comparison to same‐age peers (e.g., breast development is viewed as earlier/later/at the same time).

Confusion around status versus timing arises when examining objective timing, as objective timing and pubertal status both focus on observable physical changes. Traditionally, objective pubertal timing was assessed categorically, where more extreme deviations from a normative standard was used to create timing groups. This conceptualization rested on the assumption that individuals whose pubertal development occurred substantially earlier or later were likely to experience differential risk for psychopathology because of biological (e.g., earlier exposure to gonadal hormones) and/or psychosocial (e.g., mismatch between physical development and emotional/psychological maturity) factors (Berenbaum et al., 2015). Importantly, researchers needed to provide theoretical justification for group cut‐offs to ensure they identified youth whose development was substantially earlier/later than peers (see Dorn et al., 2006). Standard deviation (SD) cut‐offs (e.g., 1.0–2.5 SDs above/below mean age for a marker) were commonly used. For example, retrospective studies would assess age at menarche and categorize post‐pubertal girls who were 2.5 SDs below/above mean age of menarche as early/late maturers, respectively; all other girls were categorized as on time. Cross‐sectional studies during adolescence would commonly use other cut‐offs to include all adolescents rather than only those who were post‐pubertal (e.g., early maturers are ≥80th percentile of pubertal status for age; late maturers are ≤20th percentile; all others on time). Although this approach has key limitations (e.g., some on‐time youth will end up as late maturers once puberty is complete; Berenbaum et al., 2015), it provides a snapshot of the impact of pubertal timing at that time. Of course, longitudinal studies directly address this issue by characterizing trajectories of early maturers, on‐time youth who remained on time, and on‐time youth who became late maturers.

More recently, researchers used continuous, non‐categorical methods for assessing timing by taking a continuous status score (e.g., PDS score) and either standardizing it within age or regressing age out and saving the residual (Ullsperger & Nikolas, 2017). The advantage of this approach is timing can be examined in all youth, not just those in extreme groups, and statistical power is maximized with increased sample sizes and continuous rather than categorical analytic models.

## Blurring of pubertal status and timing

Despite these strengths, there are key limitations to the continuous approach. First, it changes the nature of the pubertal timing construct. As noted above, the original conceptualization focused on youth whose development differed substantially from peers (e.g., ±2.5 SDs). The continuous approach analyzes all timing differences, even those within normative developmental windows, regardless of their magnitude/clinical significance. For example, in a sample of 12‐year‐olds, the majority of youth may differ relatively minimally from each other (e.g., <1 SD difference in their pubertal timing). The extent to which there are pubertal timing differences on mental health in youth that are relatively similar to each other is an empirical question.

Second, it is difficult to separate pubertal timing from status when using the continuous approach during adolescence (Baams, Dubas, Overbeek, & van Aken, 2015; Beltz et al., 2026; Berenbaum et al., 2015).

Early maturers necessarily have more advanced pubertal status, and late maturers have less advanced status. A higher standardized score in one 12‐year‐old girl relative to another 12‐year‐old indicates that the first girl is more advanced than the second girl and more advanced than the mean pubertal status of the sample. It does not necessarily mean that the first girl is experiencing categorically different psychosocial or biological processes or a developmentally abnormal puberty trajectory. It simply states that she is more advanced. This is different than when we identify a 9‐year‐old girl whose pubertal status is several SDs more advanced than other girls her age. This girl is outside of the bounds of typical pubertal development and is very likely experiencing a different biological/psychosocial environment than other girls. Likewise, a 15‐year‐old girl who is still in early‐to‐mid‐puberty likely experiences different biological/psychosocial milieus than on‐time or post‐pubertal girls. By contrast, 15‐year‐old girls who are in late puberty but whose standardized timing scores are only modestly different likely do not experience significantly different hormonal/psychosocial environments. These more relative deviations could be important for psychopathology, but they are not the same types of deviations as categorical timing differences.

Finally, difficulties separating status from timing have led to the same measure of puberty being conceptualized as both constructs. For example, studies examining pubertal status after controlling for age have been conceptualized as studying pubertal status by some (Berenbaum et al., 2015; Dorn et al., 2006) and pubertal timing by others (Baams et al., 2015; Ullsperger & Nikolas, 2017). It is easy to see where the confusion arises. If your conceptualization of pubertal timing focuses on categorical differences between early/on‐time/late maturers, then you would think of these studies as measures of pubertal status, as they examine how being more or less advanced in pubertal status (after controlling for age) impacts outcomes. But if you think pubertal timing is any deviation in the timing of pubertal status relative to same‐age peers (no matter how large or extreme), then you would characterize these as studies of pubertal timing, as they assess how early/late an individual is relative to peers. Both views are technically correct, as the interpretation is guided by the researchers' views/theories of the nature of the pubertal timing construct. But having the same measure characterized as two different processes is problematic for our field and hinders the development of etiologic models of puberty's effects.

## Potential solutions

Importantly, we are not the first to raise these issues (Baams et al., 2015; Beltz et al., 2026; Berenbaum et al., 2015; Ullsperger & Nikolas, 2017). Others have cautioned that the confounding of status and timing should be listed as a limitation in studies using the continuous approach with an acknowledgement that findings could reflect status, timing, or both. This acknowledgement rarely happens, and we are guilty of not doing this ourselves (e.g., Klump, Culbert, O'Connor, Fowler, & Burt, 2017). One straightforward solution therefore is to acknowledge these limitations and be clearer about what we are and are not studying and what we can and cannot differentiate in our studies of puberty.

Other solutions are more challenging. The ideal approach is to conduct longitudinal studies examining pubertal status at each age and growth trajectories in clearly defined early, on‐time, and late maturing groups (Beltz et al., 2026). However, longitudinal studies are rare and becoming rarer as funding tightens. Alternatively, cross‐sectional studies could include large samples and wide age ranges (e.g., ages 7–17) to simultaneously test categorical and continuous timing variables and compare/contrast the strength of their effects.

Another complementary approach is to change the language we use for pubertal timing. When studying categorical timing across distinct groups, we propose that the traditional ‘objective pubertal timing’ and ‘early/on‐time/late maturers’ descriptors are used. By contrast, when employing the continuous approach, we propose that ‘age‐adjusted objective pubertal timing’ and ‘relatively earlier, on‐time, and later maturers’ descriptors be used. These later terms make it clear that timing is relative to same‐age peers, not a clinical/normative/statistical standard; timing is not categorically or clinically ‘early’ or ‘late’, but it is earlier, later, or similar to same‐age peers. This differentiation of language could make it significantly easier to identify studies using categorical versus continuous approaches and compare their findings. It would also make clearer that the two constructs are different and the need for researchers to think critically about their theory of timing effects and whether a categorical or continuous approach is better for addressing their research questions.

## Conclusions

Many of our points are not new. We are quite literally ‘sitting on the shoulders of giants’ (Newton, 1675, letter to Robert Hooke) like Drs. Dorn, Berenbaum, Beltz, Ullsperger, and Nikolas (among others) who previously raised several of these issues. We simply re‐emphasize the need for the field to take notice and change routine practices that confound status and timing and make it increasingly difficult to develop sophisticated models of puberty effects. We hope that our discussion and recommendations encourage researchers to think critically about their theories and optimally design their studies to differentiate pubertal processes in this critical area of adolescent health.
